# Editorial: The application of multi-omics analysis in translational medicine

**DOI:** 10.3389/fmed.2025.1744814

**Published:** 2025-12-04

**Authors:** Haihui Huang, Madhu Chetty

**Affiliations:** 1 Shaoguan Research Center of the National Engineering Laboratory for Big Data System Computing Technology, Shaoguan University, Shaoguan, China; 2 Guangdong Provincial Laboratory of Traditional Chinese Medicine, Hengqin, China; 3 Institute of Innovation, Science and Sustainability, Federation University Australia, Ballarat, VIC, Australia

**Keywords:** multi-omics analysis, translational medicine, data integration, personalized medicine, computational biology

The primary goal of translational medicine is to convert fundamental biological discoveries into tangible improvements in human health. Achieving this requires a holistic understanding of the complex molecular networks governing disease. Multi-omics analysis has become an essential paradigm in this endeavor, integrating data from diverse layers such as genomics, transcriptomics, proteomics, and metabolomics. This integration is crucial for bridging the gap between basic research and clinical application, facilitating precise diagnostics and personalized therapies.

However, the application of multi-omics approaches faces challenges, including the complexity of data integration, the interpretation of high dimensional datasets, and the standardization required for clinical implementation. This Research Topic, “*The Application of Multi-omics Analysis in Translational Medicine*”, presents 14 articles that navigate these challenges and showcase advancements in this rapidly evolving field. The Research Topic underscores the power of integrative strategies across a spectrum of methodological innovations and complex diseases.

## Advancing computational methodologies and data integration

The volume and complexity of multi-omics data demand sophisticated computational tools. A significant focus within this Research Topic is the development of artificial intelligence (AI) and machine learning frameworks, alongside the integration of diverse data types, to enhance predictive accuracy.

Predicting drug response is central to precision oncology. Miao et al. introduced an innovative drug response prediction model (NMDP) to address challenges in feature extraction and data fusion. Their model utilizes an interpretable semi supervised weighted SPCA module and integrates convolution methods with Kolmogorov Arnold Networks, demonstrating superior performance in predicting drug sensitivity.

Prioritizing actionable drug targets from vast genomic landscapes remains a significant hurdle. Gu and Chen developed GETgene AI, a framework that combines network-based prioritization, machine learning, and automated literature analysis powered by advanced language models. Applied to pancreatic cancer, GETgene AI successfully prioritized high priority targets, illustrating how AI driven approaches can accelerate drug discovery.

The integration of molecular data with imaging modalities represents another critical frontier. Huang Y. et al. explored the predictive potential of quantitative histopathological image features (HIF) in glioblastoma. By integrating HIF with genomics, transcriptomics, and proteomics, they found that the integrated multi-omics model significantly enhanced prognostic accuracy compared to single omics approaches.

Similarly, Li et al. developed a radiomics model for predicting chemoradiotherapy response in advanced non-small cell lung cancer. They integrated radiomic features from both the primary lesion and nodal disease with clinical data. This multimodal composite model demonstrated superior predictive performance, emphasizing the value of comprehensive data integration in clinical decision making.

## Multi-omics insights into molecular mechanisms of oncological and chronic diseases

Multi-omics research continues to deepen our understanding of tumorigenesis, classification, and the interplay with systemic conditions and chronic diseases.

Loganathan and Doss investigated the interconnected molecular mechanisms between breast cancer and diabetes. Utilizing transcriptomic and exomic analyses across different cohorts, they identified shared pathways related to extracellular matrix organization and immune regulation. Their analysis highlighted the TNF pathway as a central link connecting chronic inflammation, insulin resistance, and tumor growth.

Pugazenthi et al. provided a review of the application of multi-omics analysis for pituitary neuroendocrine tumors (PitNETs). They summarized how integrated approaches have contributed to a deeper understanding of PitNET pathogenesis, revealing molecular subtypes and regulatory networks that inform classification and advance personalized medicine.

The power of multi-omics analysis extends to degenerative and inflammatory diseases. Zhang et al. aimed to identify novel risk genes for intervertebral disc disorder by integrating large scale multi-omics analyses, including transcriptome wide association studies and proteome wide association studies. Their integrative analysis and experimental validation confirmed the pathogenic roles of TMEM190, CILP2, and FOXO3, highlighting CILP2 as a potential druggable target.

Jin et al. focused on periodontitis by integrating transcriptomic and DNA methylation profiles. Their analysis explored the immune microenvironment and utilized machine learning to identify nine key diagnostic biomarkers. Subsequent network pharmacology analysis identified potential targeted drugs, offering new therapeutic avenues.

## The nexus of microbiome, metabolism, and host response

The integration of microbiome and metabolomic data with host multi-omics profiles is rapidly emerging as a critical area of translational research, revealing intricate interactions between host metabolism, immune function, and microbial communities.

The connection between gut microbiota and systemic disease is an area of intense investigation. Liu L. et al. reviewed the emerging evidence surrounding the gut microbiota lung axis in lung cancer. They synthesized data indicating that gut dysbiosis is associated with worse prognosis and impacts the efficacy of immune checkpoint blockade, suggesting potential adjunctive therapeutic strategies through microbiome modulation.

The microbiome's role extends to neuroscience. Wang et al. employed a multi-omics approach to unravel the mechanisms of propofol induced psychological dependence. By integrating transcriptomics, metabolomics, and gut microbiome sequencing in a mouse model, they identified significant changes in neuroactive ligand receptor interaction pathways and gut microbial composition, suggesting a complex bidirectional signaling mechanism.

In the context of musculoskeletal health, Liu Y. et al. conducted an integrative analysis of serum microorganisms and serum metabolomics in osteoporosis patients. Their findings revealed distinct microbial compositions and significant differences in lipid metabolism pathways associated with osteoporosis, providing candidate biomarkers for early diagnosis.

Metabolomics also proved valuable in elucidating the mechanisms of traditional therapies. Sun et al. investigated the protective mechanisms of Angelica sinensis polysaccharide (ASP) against recurrent spontaneous abortion. Through metabolomic analysis and assessment of autophagy levels, they found that ASP restores diminished autophagy activity and regulates key metabolic pathways, including glycolysis/gluconeogenesis.

## Emerging modalities and perspectives

The Research Topic also highlights the potential of novel biological entities and advanced analytical modalities. The diagnostic and therapeutic potential of exosomes was reviewed by Odehnalová et al.. As carriers of disease specific biomarkers, these extracellular vesicles offer opportunities for non-invasive detection, targeted drug delivery, and regenerative medicine in cancer and neurodegenerative diseases.

Furthermore, Huang F. F. et al. provided neuroimaging evidence for the central mechanisms of acupuncture in non-specific low back pain through a systematic review and meta-analysis. Utilizing functional neuroimaging data, this study demonstrated that acupuncture modulates pain processing through the insula and limbic system, validating its clinical efficacy and exploring its underlying mechanisms.

The studies compiled in this Research Topic collectively demonstrate the profound impact of multi-omics analysis on translational medicine. By embracing integrative approaches, novel computational methods, and the inclusion of diverse data types such as microbiome profiles and imaging features, these investigations are significantly advancing the field. The insights generated here not only enhance our understanding of complex diseases but also pave the way for more precise diagnostics and personalized therapeutic strategies, bringing us closer to the realization of personalized healthcare.

